# Integrin-Linked Kinase Regulates Interphase and Mitotic Microtubule Dynamics

**DOI:** 10.1371/journal.pone.0053702

**Published:** 2013-01-21

**Authors:** Simin Lim, Eiko Kawamura, Andrew B. Fielding, Mykola Maydan, Shoukat Dedhar

**Affiliations:** 1 Department of Integrative Oncology, British Columbia Cancer Research Centre, Vancouver, British Columbia, Canada; 2 Interdisciplinary Oncology, Faculty of Graduate Studies, University of British Columbia, Vancouver, British Columbia, Canada; 3 Department of Cellular and Molecular Physiology, University of Liverpool, Liverpool, Merseyside, United Kingdom; 4 Department of Biochemistry and Molecular Biology, Life Sciences Institute, University of British Columbia, Vancouver, British Columbia, Canada; Emory University, United States of America

## Abstract

Integrin-linked kinase (ILK) localizes to both focal adhesions and centrosomes in distinct multiprotein complexes. Its dual function as a kinase and scaffolding protein has been well characterized at focal adhesions, where it regulates integrin-mediated cell adhesion, spreading, migration and signaling. At the centrosomes, ILK regulates mitotic spindle organization and centrosome clustering. Our previous study showed various spindle defects after ILK knockdown or inhibition that suggested alteration in microtubule dynamics. Since ILK expression is frequently elevated in many cancer types, we investigated the effects of ILK overexpression on microtubule dynamics. We show here that overexpressing ILK in HeLa cells was associated with a shorter duration of mitosis and decreased sensitivity to paclitaxel, a chemotherapeutic agent that suppresses microtubule dynamics. Measurement of interphase microtubule dynamics revealed that ILK overexpression favored microtubule depolymerization, suggesting that microtubule destabilization could be the mechanism behind the decreased sensitivity to paclitaxel, which is known to stabilize microtubules. Conversely, the use of a small molecule inhibitor selective against ILK, QLT-0267, resulted in suppressed microtubule dynamics, demonstrating a new mechanism of action for this compound. We further show that treatment of HeLa cells with QLT-0267 resulted in higher inter-centromere tension in aligned chromosomes during mitosis, slower microtubule regrowth after cold depolymerization and the presence of a more stable population of spindle microtubules. These results demonstrate that ILK regulates microtubule dynamics in both interphase and mitotic cells.

## Introduction

Integrin-linked kinase (ILK) was first identified in 1996 as an interactor of β1- and β3-integrin subunit cytoplasmic domains [1]. It localizes to the focal adhesions [2] where it functions both as a serine-threonine kinase and an adaptor protein in a multiprotein complex to relay extracellular signals from the integrins and growth factors to the cell interior [3]. Studies have demonstrated a role for ILK in regulating cell survival, proliferation, angiogenesis and PI3 kinase-dependent signaling [4]. Through its interactions with focal adhesion proteins α- and β-parvin, PINCH and paxillin, ILK also regulates actin cytoskeleton organization, cell adhesion, spreading and migration [3]. Several gene knockout studies have since revealed essential roles of ILK in embryonic development, tissue homeostasis, and organ function, while ILK levels have been shown to be elevated in many cancer types and correlates with poor prognosis [5]. Indeed, ILK appears to be differentially required for growth and survival between normal and cancer cells [6].

The pharmacological inhibition of ILK activity has been pursued with the development of small-molecule inhibitors against ILK's kinase activity. One of these inhibitors, QLT-0267, has previously been described to be highly selective against ILK phosphotransferase activity [7]. In vitro, QLT-0267 inhibited the kinase activity of purified ILK in an ATP-competitive manner and showed the most potent inhibition when compared with several other kinase inhibitors [8]. In cells, this compound inhibited ILK kinase activity with a half maximal inhibitory concentration (IC_50_) of between 2 and 5 µM, depending on cell type [6].

More recently, ILK was discovered to also bind tubulin [9] and localize to the centrosome, where it organizes the mitotic spindle [10], and also regulates centrosome clustering [11]. Here, it resides in a different multiprotein complex, interacting with a distinct set of binding partners, including colonic and hepatic tumor over-expressed gene (ch-TOG) and RuvB-like 1 (RUVBL1). We have previously shown that ILK is required for the formation of a protein complex between Aurora-A and ch-TOG or transforming, acidic coiled-coil containing protein 3 (TACC3), but not their localization to the centrosome [10]. Additionally, ILK regulates TACC3 Ser558 phosphorylation in an Aurora-A-dependent manner [11]. These three proteins, Aurora-A, ch-TOG and TACC3, are all key regulators of mitotic spindle organization [9], [12], [13].

Proper mitotic spindle organization and microtubule dynamics are essential for the successful alignment and segregation of duplicated chromosomes during mitosis. Microtubules undergo dynamic transitions between growth and shrinkage, known as dynamic instability. This process is now recognized to be essential for progression through mitosis [14]. Several microtubule-targeted drugs, including paclitaxel, are known to suppress microtubule dynamic instability and induce disorganized mitotic spindles and mitotic arrest [15]–[19].

Our previous study demonstrated that pharmacological inhibition or depletion of ILK resulted in a disorganized mitotic spindle phenotype [10] that resembled paclitaxel-treated spindles, and suggested that ILK may regulate microtubule dynamics. Given that elevated ILK expression is common in many cancer types, we investigated effects of ILK overexpression on microtubule dynamics. We show here that overexpressing ILK in HeLa cells resulted in a shorter duration of mitosis and decrease in sensitivity to paclitaxel, a chemotherapeutic agent that suppresses microtubule dynamics. Measurement of interphase microtubule dynamics revealed destabilization of microtubules when ILK was overexpressed in HeLa cells. This suggests that microtubule depolymerization resulting from excess ILK may counteract the stabilizing activity of paclitaxel, explaining the observed decrease in sensitivity to this agent. Conversely, the use of QLT-0267 resulted in suppressed microtubule dynamics, demonstrating a new mechanism of action for this compound. We further show that treatment of HeLa cells with QLT-0267 resulted in higher inter-centromere tension in aligned chromosomes during mitosis, slower microtubule regrowth after cold depolymerization and the presence of a more stable population of spindle microtubules.

## Results and Discussion

### Overexpression of ILK shortens duration of mitosis

**Figure 1:**
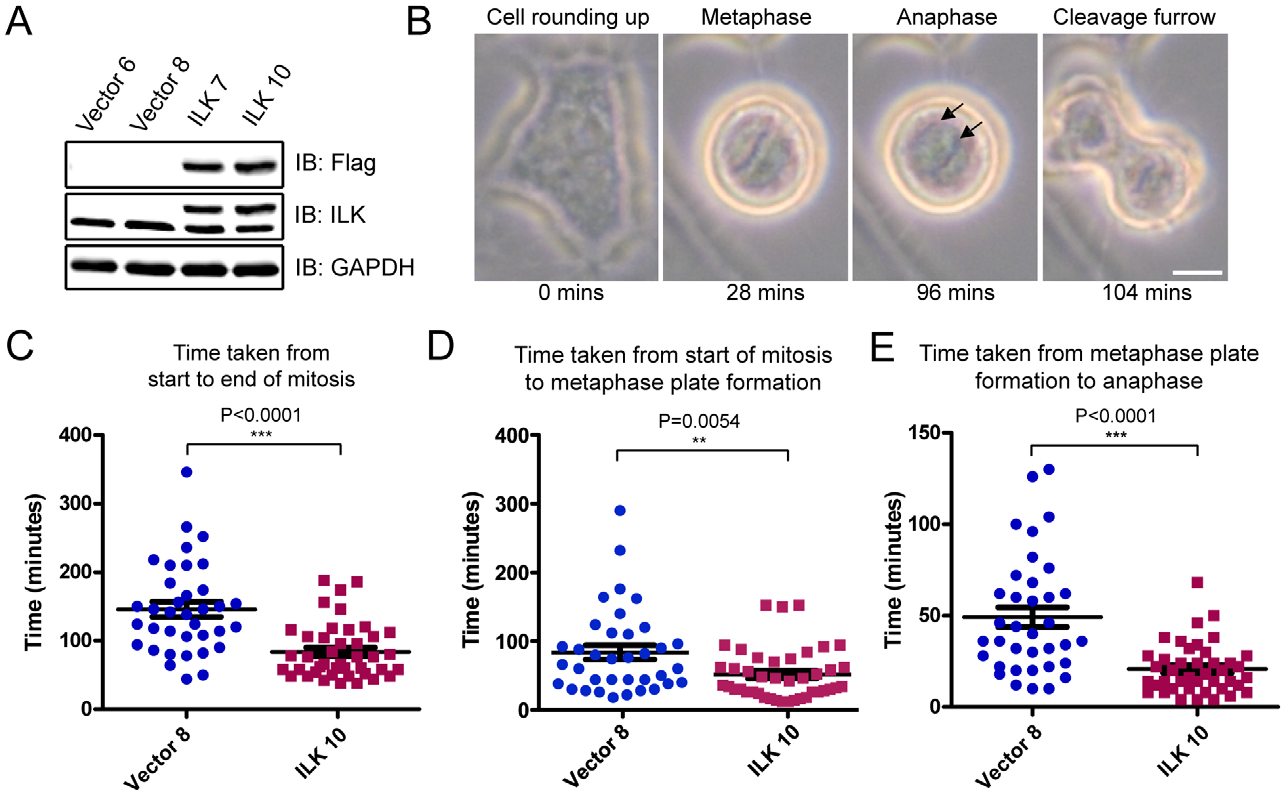
ILK overexpression shortens duration of mitosis in HeLa cells. (A) Immunoblot analysis of HeLa cell clones stably expressing control vector (clones Vector 6 and Vector 8), and the epitope-tagged Flag-ILK (clones ILK 7 and ILK 10). (B) Representative time-lapse images (taken every 2 minutes) of HeLa clone Vector 8. Images show the four stages of mitosis used for measurement of time required for each phase of mitosis: cell rounding up (start of mitosis), metaphase, onset of anaphase (arrows indicate separating chromosomes), and formation of cleavage furrow (end of mitosis). Bar = 10 µm. (C–E) Scatter plot of time taken by vector control HeLa cells (Vector 8, N = 36) and ILK-overexpressing HeLa cells (ILK 10, N = 42) to complete the various stages of mitosis. Results represent mean ± S.E.M.

We previously reported that knockdown and pharmacological inhibition of ILK by QLT-0267 resulted in an increased mitotic index [10]. To test whether overexpression of ILK would have the opposite effect by shortening mitotic progression, we generated HeLa cell clones that overexpressed FLAG-tagged ILK by lentiviral transduction. Four single cell clones were selected for subsequent experiments - Vector 6 and Vector 8 as vector controls, and ILK 7 and ILK 10 as ILK-overexpressing clones (Figure 1A). Immunofluorescence analysis was performed to confirm the localization of Flag-ILK to the focal adhesions (Figure S1A), and western blot analysis showed the presence of Flag-ILK in the mitotic spindle fraction (Figure S1B). The morphology of ILK 7 and ILK 10 cells appeared to be slightly more elongated and mesenchymal compared to Vector 6 and Vector 8 cells, consistent with an activation of epithelial-mesenchymal transition [20], [21] (Figure S1C). The control cells (Vector 6 and Vector 8) retained an epithelial-like morphology resembling that of the parental HeLa cells. These results show that functional Flag-ILK proteins were expressed in ILK 7 and ILK 10 cells.

To investigate the effect of ILK overexpression on mitosis, we monitored mitotic progression by time-lapse imaging in two of the stable clones, Vector 8 and ILK 10 (Movies S1 and S2, which are provided as supporting information). Time-lapse images were obtained every 2 minutes (Figure 1B), and the time spent to progress through each stage of mitosis was measured (details in Materials and Methods) (Figures 1C–E).

Detailed analysis of the time-lapse images revealed that ILK overexpression shortened the duration of mitosis from 145±10.9 minutes to 84.0±6.24 minutes (mean ± S.E.M., P<0.0001) (Figure 1C). The time taken to complete prophase and prometaphase was reduced in ILK overexpressing cells (from 83.8±10.2 minutes in control cells to 51.4±5.74 minutes in ILK overexpressing cells; mean ± S.E.M., P = 0.0054) (Figure 1D). The time taken from metaphase to anaphase onset was also shorter (49.2±5.36 minutes in control cells and 20.8±2.11 in ILK overexpressing cells; mean ± S.E.M., P<0.0001) (Figure 1E).

Our finding that ILK overexpression reduces the duration of mitosis is consistent with our previous report that ILK knockdown and pharmacological inhibition with QLT-0267 arrest cells in mitosis. Given the importance of microtubule dynamics to all stages of mitosis, one of the possible reasons for our observations could be the alteration of microtubule dynamics due to ILK overexpression. It should be noted that our observations of a shorter metaphase-anaphase duration could also be attributed to a weakened spindle assembly checkpoint, likely causing the cells to make errors in chromosome attachment and segregation, and display some chromosome instability. While we do not rule out this possibility, for this study we decided to further investigate whether ILK plays a role in regulating microtubule dynamics.

### Overexpression of ILK is associated with decreased paclitaxel sensitivity

ILK expression is often elevated in many cancer types [5]. Since altered microtubule dynamics could be one of the reasons for faster mitosis in ILK overexpressing HeLa cells, we asked if ILK overexpression would also alter sensitivity to drugs that target microtubule dynamics. Therefore we tested the effect of ILK overexpression on sensitivity to paclitaxel (Taxol). Paclitaxel and related taxanes are commonly used in the treatment of a range of epithelial cancers [22] and inhibit mitosis by suppressing microtubule dynamics [17], [19].

**Figure 2:**
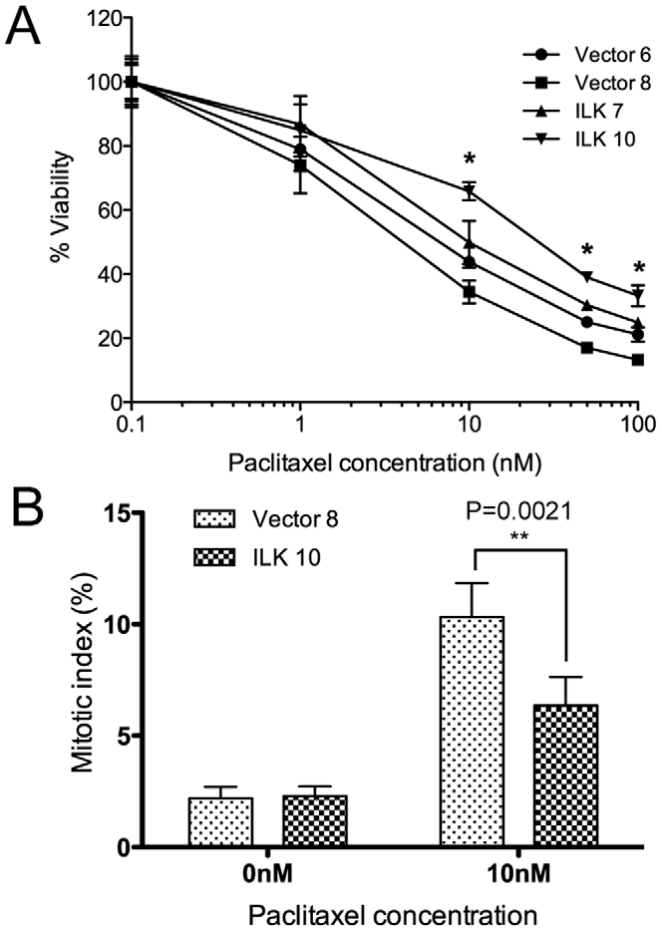
ILK overexpression in HeLa cells is associated with decreased sensitivity to paclitaxel. (A) Percentage viability of HeLa clones (control: Vector 6 and Vector 8; ILK overexpressing: ILK 7 and ILK 10) after various concentrations of paclitaxel treatment for 48 hours. Results indicate mean ± S.D., and are representative of 3 independent experiments. An asterisk (*) indicates significant difference (P<0.01) between each of the control and overexpressing cell lines. (B) ILK overexpression dampens the paclitaxel-induced increase in mitotic arrest in HeLa cells. The mitotic indices of control Vector 8 and ILK-overexpressing ILK 10 were quantified after treatment with DMSO or 10 nM paclitaxel for 48 hours. Bar graph shows mean ± S.E.M., N>1000 cells.

A cell viability test was carried out using the MTT ((3-(4,5-Dimethylthiazol-2-yl)-2,5-diphenyltetrazolium bromide) assay. Cells were exposed to a range of paclitaxel concentrations for 48 hours, and then the MTT assay was carried out to determine the percentage of viable cells. After 48 hours of paclitaxel treatment, the ILK overexpressing cell lines, ILK 7 and ILK 10, showed decreased sensitivity to paclitaxel (IC_50_ 10 nM and 25 nM, respectively), when compared to the controls, Vector 6 (IC_50_ 6.5 nM) and Vector 8 (IC_50_ 4 nM) (Figure 2A). The IC_50_ fold difference ranges from 1.5-fold (between Vector 6 and ILK &7) to 6.3-fold difference (between Vector 8 and ILK 10). While the changes to paclitaxel sensitivity were modest, likely due to the low levels of exogenous ILK expression relative to endogenous ILK (Figure 1A), the differences between each of the four cell lines, as determined by the Student's t-test (P<0.01) (Table S1), were significant at paclitaxel concentrations of 10 nM, 50 nM and 100 nM (indicated by an asterisk in Figure 2A).

To further examine the effect of paclitaxel in ILK overexpressing cells, we investigated another known paclitaxel effect – mitotic arrest due to perturbation of microtubule dynamics. The mitotic index was determined for Vector 8 and ILK 10 cells treated with DMSO or 10 nM paclitaxel for 48 hours. To detect mitosis, cells were immunostained with anti-α-tubulin to visualize mitotic spindles (Figure 2B). Without paclitaxel treatment, both cell lines had comparable mitotic indices at 2.2% for Vector 8 and 2.3% for ILK 10. As expected, when vector control cells were subjected to 10 nM paclitaxel, the mitotic index increased to 10.3%, but this increase was much more subdued in the ILK overexpressing cell line at only 6.36% (P = 0.0021), suggesting that the effect of paclitaxel was reduced in these cells. It should be noted that HeLa cells have a doubling time of approximately 24 hours, and the mitotic indices were nowhere near 100%, likely due to mitotic slippage. Our findings of higher IC_50_ values in the MTT assay and a lower paclitaxel-induced mitotic arrest indicate that ILK overexpression contributes to decreased paclitaxel sensitivity in HeLa cells.

There are a number of possible reasons for the lowered paclitaxel sensitivity as a result of ILK overexpression. One of them could be the override of the spindle assembly checkpoint, as was seen previously in Aurora A overexpressing HeLa cells [23]. Secondly, the activation of survival pathways such as PI3k/AKT is known to confer resistance to paclitaxel [24], and ILK regulates cell survival through phosphorylation and activation of AKT [25]. A third explanation could be the alteration of microtubule dynamics in a manner that counteracts the effects of paclitaxel, which we pursued next.

### ILK regulates microtubule dynamic instability in interphase HeLa cells

**Figure 3:**
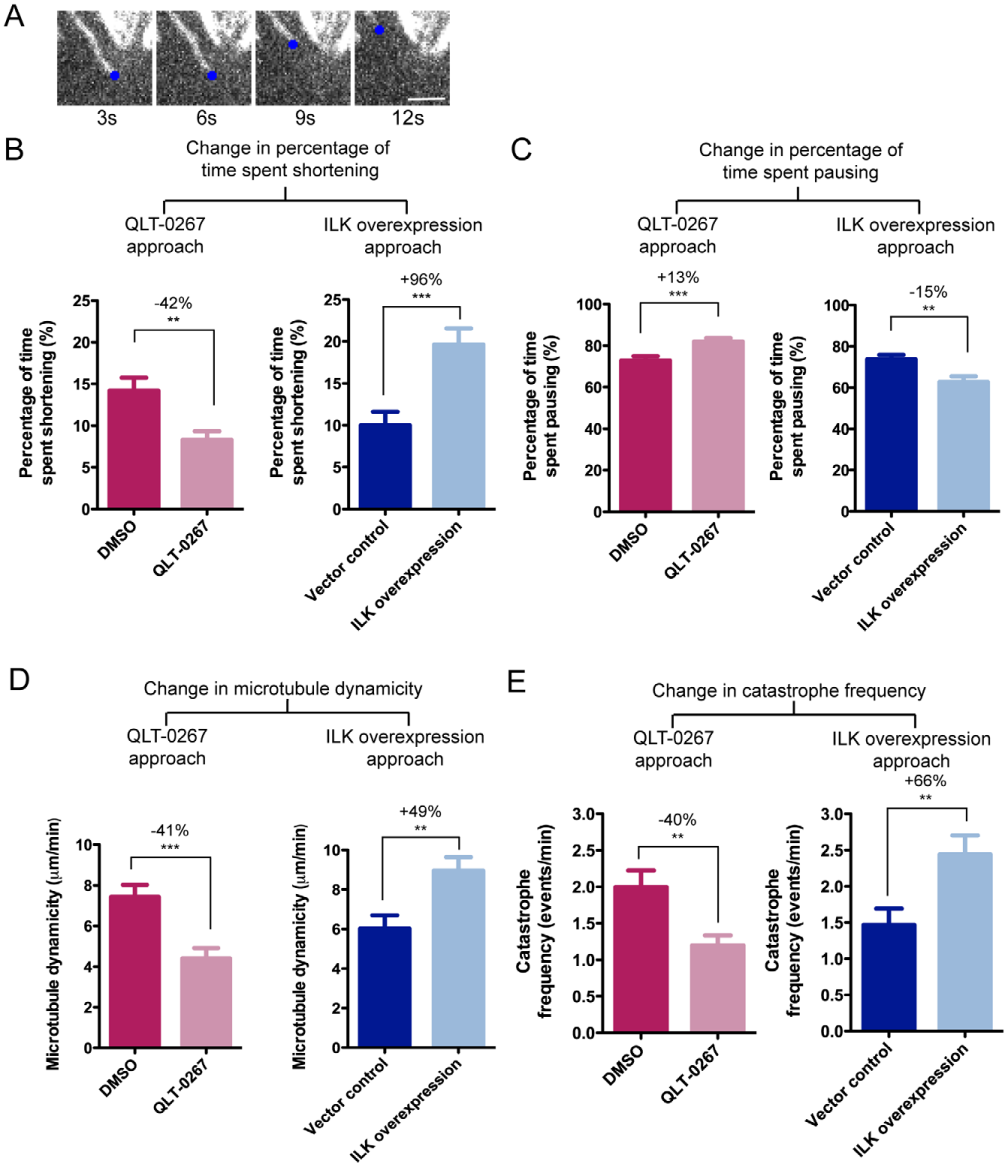
ILK overexpression affects microtubule dynamic instability in living interphase HeLa cells. (A) Time-lapse images of microtubule ends in living ILK-overexpressing HeLa clone ILK 10. Images were obtained every 3 s to track the movement of microtubule ends. This series of representative images shows a microtubule end (tracked by a blue dot) pausing from 3 s to 6 s, then shortening from 6 s to 12 s. Bar = 2 µm. (B–D) ILK inhibition suppresses, while ILK overexpression increases microtubule dynamic instability. HeLa clones Vector 8 and ILK 10 were stably transfected with tubulin-venus. The rates and parameters of microtubule dynamic instability were compared between Vector control (Vector 8) and ILK overexpression (ILK 10), as well as between DMSO- or QLT-0267-treated Vector 8 cells. Bar graphs show mean ± S.E.M., N = 75 (DMSO), 77 (QLT-0267), 54 (Vector control), 46 (ILK overexpression).

To directly address the effect of ILK overexpression on microtubule dynamics, we measured the parameters of microtubule dynamic instability in living HeLa cells. Tubulin-venus was stably introduced into the cell lines Vector 8 and ILK 10 to visualize fluorescent microtubules, and live cell imaging was performed under a spinning disc confocal microscope. Since microtubules in the mitotic spindle were too dense for visualization of individual microtubules, we tracked the individual microtubule ends in the lamellar region of interphase cells (Figure 3A, and Movies S3, S4, S5 and S6). Images were taken every 3 seconds for up to 3 minutes. To complement the overexpression of ILK, we also utilized an ILK selective inhibitor, QLT-0267, to measure microtubule dynamic instability. We examined (1) the effect of ILK overexpression by comparing untreated Vector 8 and ILK 10 cells and (2) the effect of ILK inhibition by comparing DMSO and QLT-0267-treated Vector 8 cells.

Microtubules are intrinsically dynamic polymers that exhibit dynamic instability – a behavior in which microtubule ends undergo frequent transitions between growth and shrinkage. After tracking and measuring the distance traveled by individual microtubule ends, the parameters of microtubule dynamic instability were calculated (see Materials and Methods for details) and tabulated (Tables S2, S3, S4). Four of the parameters (% time spent shortening, % time spent pausing, dynamicity and time-based catastrophe frequency) were significantly altered and exhibited opposite effects when ILK was inhibited or overexpressed (Figures 3B–E). With QLT-0267 treatment, microtubules spent 42% less time shortening and 13% more time pausing, while the opposite was observed with ILK overexpression – 96% more time spent shortening, and 15% less time pausing (Figs. 3B, C). Microtubule dynamicity reflects the total length grown or shortened at the microtubule ends and is a measure of overall tubulin turnover. This parameter was suppressed by 41% with QLT-0267 treatment, but became elevated by 49% after ILK overexpression (Fig. 3D). Similarly, ILK inhibition with QLT-0267 reduced the time-based catastrophe frequency (the frequency of transition from growing or pausing, to shortening phases, over time) by 40%, while ILK overexpression increased the catastrophe frequency by 66%.

Interestingly, the rate of growth was not affected by ILK overexpression nor QLT-0267 treatment (Tables S2 and S3). ILK overexpressing cells showed a faster rate of shortening, while QLT-0267 treatment appeared to slightly reduce the shortening rate, although the change was not statistically significant. Overall, overexpression of ILK favors microtubule destabilization, by increasing shortening rate, time spent for shortening and catastrophe events, and inhibition of ILK by QLT-0267 exhibits opposite effects while stabilizing microtubules. Taken together, these results demonstrate that ILK plays a role in regulation of interphase microtubule dynamics.

A recent study made the interesting observation that ILK could regulate the local microtubule dynamics required for plasma membrane targeting of caveolae in mouse keratinocytes [26]. ILK was found to recruit the scaffold protein IQGAP1 and its downstream effector mDIA1 to nascent, cortical adhesion sites, thereby inducing local stabilization of microtubules, which is required for the proper trafficking of caveolin-1-containing vesicles. The same study by Wickström *et al.* also measured microtubule dynamics in interphase mouse keratinocytes after ILK gene deletion (ILK-K5) and found an increase in catastrophe and rescue frequencies, as well as a decrease in pausing duration, suggesting microtubule destabilization. Interestingly, not all the parameters of microtubule dynamics in ILK-K5 reflect our data for ILK overexpression or QLT-0267 treatment. This may be because the expected effects of gene knockdown are not necessarily analogous to kinase inhibition or indeed opposite to that expected for protein overexpression. However, it also suggests that ILK's regulation of microtubule dynamics may be dependent on cell type, species and/or even disease state (e.g. normal vs. cancer). For example, caveolae are only abundant in specific cell types such as the smooth muscle cells, fibroblasts, endothelial cells and adipocytes [27], and cells that do not utilize as many caveolae may have markedly different regulation of microtubule dynamics at the cell periphery.

The targeting of microtubule dynamics is a common strategy in chemotherapy, and the effects of paclitaxel on microtubule dynamics have been well studied both in isolated microtubules and living cells [17], [19]. The effects of QLT-0267 on microtubule dynamics seen here are similar to that reported for paclitaxel – reduced dynamicity and an increase in distance-based rescue frequency [19]. Paclitaxel is also known to reduce the rate of microtubule shortening [19], a parameter that was increased by ILK overexpression, in addition to elevated microtubule dynamicity. This raised the question of whether ILK overexpression influences microtubule dynamics in a way that would counter paclitaxel's effects and alter drug sensitivity. Increased microtubule dynamics have been associated with paclitaxel resistance [28]. Microtubules in paclitaxel-resistant cell lines exhibit increased shortening rates, shorter percentage time pausing, and higher microtubule dynamicity [28], all of which were also observed in the ILK overexpressing cells. Indeed, we have shown that ILK overexpressing cells are less sensitive to paclitaxel. Of note, the spindle structure of ILK overexpressing cells appeared normal with proper chromosome alignment (Figure S2), showing that any changes in microtubule dynamics are not indirectly reflecting changes in spindle structure.

Alteration of microtubule dynamics thus appears to be one of the mechanisms by which ILK overexpression reduces paclitaxel sensitivity. This has important clinical implications because elevated ILK levels have been found in many cancer types [5]. We would then expect inhibition of ILK activity by QLT-0267 to complement paclitaxel treatment, but one study that examined cell viability after combination therapy with paclitaxel and QLT-0267 on breast cancer cell lines reported the two drugs to be antagonistic, although treatment with QLT-0267 and docetaxel (a synthetic analogue of paclitaxel) proved to be synergistic [29]. Given the multiple functions of ILK, it is certainly possible that the perturbation of other important ILK-regulated signaling pathways by QLT-0267 had come into play. Nevertheless, it will be useful to further study relationships between ILK expression and paclitaxel resistance in clinical samples, and to explore alternative drug therapies that consider enhanced microtubule dynamics induced by elevated ILK expression.

### Treatment of HeLa cells with QLT-0267 increases tension across sister centromeres, slows microtubule regrowth and increases stability of microtubules in mitosis

**Figure 4:**
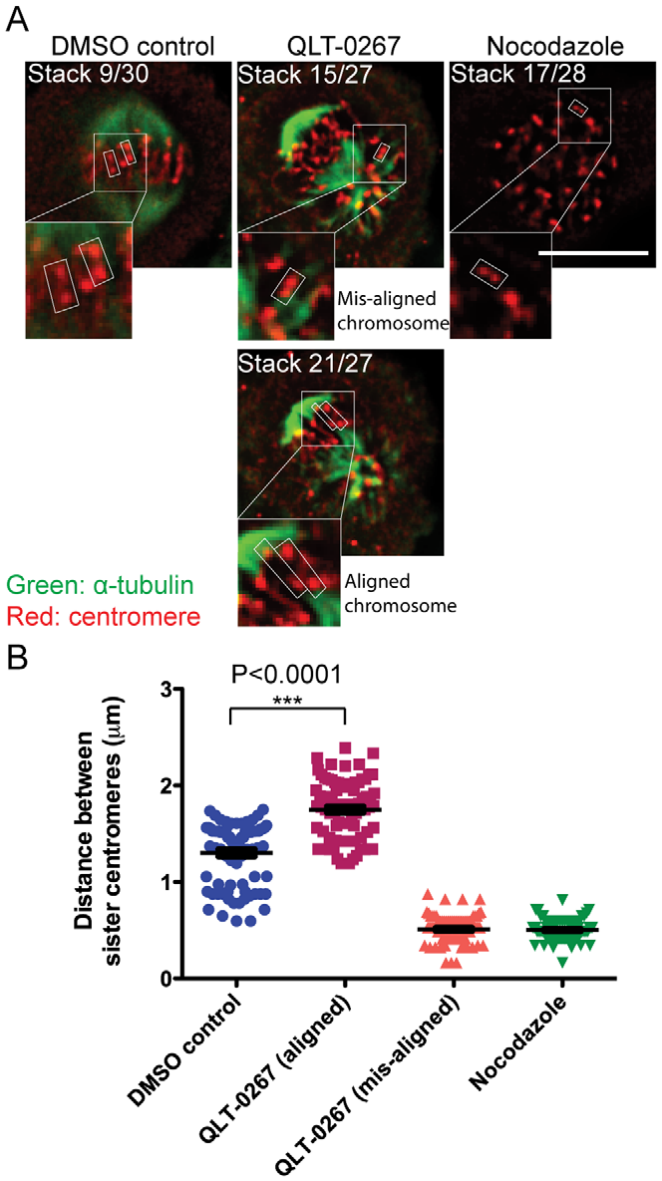
QLT-0267 treatment of HeLa cells increases sister centromere tension. (A) Representative immunofluorescence images of mitotic HeLa cells showing α-tubulin (green) and centromere (red). Cells were treated with DMSO, QLT-0267 for 6 hours or 1 µM nocodazole for 16 hours. Images were acquired as z-stacks with 0.25 µm spacing, and individual stacks were browsed through to identify sister centromeres. Small white boxes indicate examples of identifiable sister centromere pairs used for distance measurement between them. Bar = 10 µm. (B) Quantification of distance between sister centromeres in mitotic HeLa cells treated with DMSO, QLT-0267 (aligned chromosomes between both spindle poles and mis-aligned chromosomes on the side of only one pole) and nocodazole (basal control for lack of tension between sister centromeres). Results represent mean ± S.E.M. (DMSO control N = 81, QLT-0267 (aligned) N = 84, QLT-0267 (mis-aligned) N = 84, nocodazole N = 85) from 2 independent experiments.

Preclinical studies have begun to assess the use of QLT-0267 for cancer therapy [29]–[31] and since QLT-0267 was shown here to suppress interphase microtubule dynamics, we next examined the effects of QLT-0267 on mitotic microtubule dynamics. We have previously reported that ILK inhibition by QLT-0267 disrupted mitotic spindle organization and prevented the proper alignment of chromosomes to the metaphase plate in HeLa cells [10]. We first examined inter-centromere tension generated by the spindle microtubules by measuring the distance between sister centromeres [32]–[34]. HeLa cells were treated with DMSO, nocodazole or QLT-0267, then fixed and stained with anti-α-tubulin and anti-centromere (Figure 4A). Nocodazole depolymerizes microtubules and the absence of a mitotic spindle allows the measurement of a basal distance between sister centromeres that were under no tension. Z-stacks were acquired at 0.25 µm steps, and were examined to identify pairs of sister centromeres (Figure 4A, arrowed). The inter-centromere distance of both aligned and mis-aligned chromosomes in QLT-067-treated cells were quantified three dimensionally using the Z-stacks (Figure 4B). All QLT-0267-treated cells contained misaligned chromosomes of either one or both poles, confirming spindle defects previously reported [10]. Spindles typically showed asymmetrical localization of microtubules to the spindle poles (Figure 4A). The quantification of inter-centromere distance in control cells was performed only in metaphase cells that had all their chromosomes aligned between both spindle poles.

Our quantitative analysis revealed that within aligned chromosomes, the tension in QLT-0267-treated cells was greater (1.75±0.03 µm, mean ± S.E.M.) than in control cells (1.30±0.04 µm, mean ± S.E.M.). As predicted, the tension was reduced in mis-aligned chromosomes of QLT-0267 treated cells compared to aligned chromosomes of untreated cells. The inter-centromere distances of mis-aligned chromosomes in QLT-0267-treated cells were similar to the unattached chromosomes in nocodazole-treated cells, suggesting a lack of tension across the mis-aligned chromosomes. Since it has been reported that microtubule dynamics, rather than microtubule motors, could be primarily responsible for centromere stretching and relaxation [35], our data suggest that QLT-0267 treatment could also affect microtubule dynamics during mitosis.

**Figure 5:**
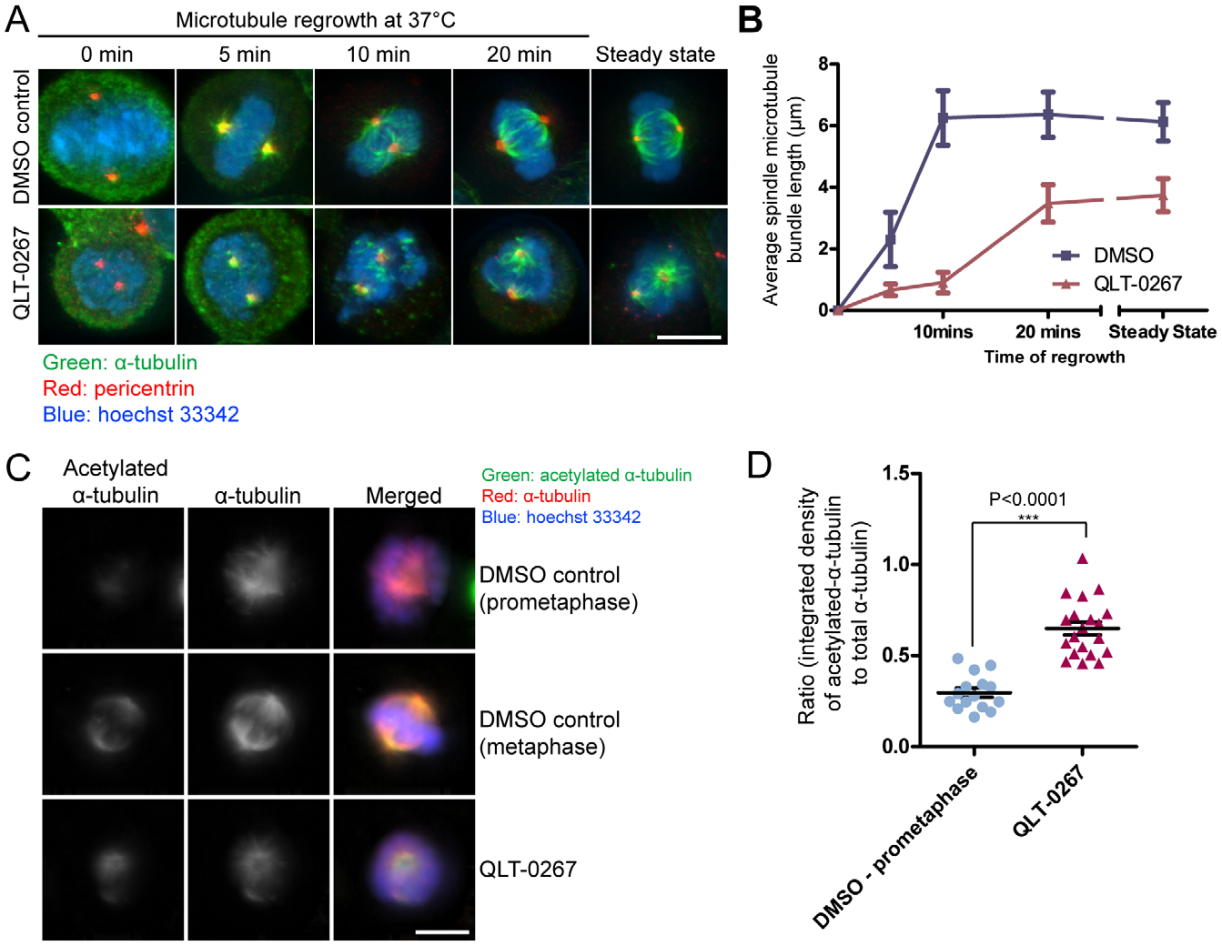
QLT-0267 treatment affects microtubule dynamics in HeLa cells during mitosis. (A) QLT-0267 treatment retards the rate of microtubule regrowth during mitosis. Representative immunofluorescence images of mitotic HeLa cells stained for α-tubulin (green), centrosome (red) and chromosomes (blue). HeLa cells were treated with DMSO or QLT-0267 for 5 hours, then chilled at 4°C for 1 hour to depolymerize microtubules, before being returned to 37°C for various time-points followed by methanol fixation. Steady state: un-chilled cells. Bar = 10 µm. (B) Quantification of microtubule length at different time-points of microtubule regrowth. Results represent mean ± S.D., N = 40 (4 longest microtubules per cell, 10 cells per condition). (C) ILK inhibition increases microtubule stability during mitosis. Representative images of HeLa cells treated with DMSO or QLT-0267 were stained for acetylated α-tubulin (green), total α-tubulin (red) and chromosomes (blue). QLT-0267-treated cells show a higher proportion of acetylated α-tubulin relative to the total amount of α-tubulin when compared to normal prometaphase cells. Bar = 10 µm. (D) Quantification of the integrated density of acetylated α-tubulin relative to total α-tubulin. Results represent mean ± S.E.M., N = 15 (DMSO prometaphase), 20 (DMSO metaphase), 20 (QLT-0267).

Next, we examined spindle microtubule regrowth after cold depolymerization to investigate QLT-0267's effects on spindle microtubule assembly. Control and QLT-0267-treated HeLa cells were put on ice at 4°C for 1 hour to completely depolymerize all microtubules, and then incubated at 37°C for varying time periods prior to fixation. Cells were subjected to immunofluorescence staining to visualize spindle microtubules and centrosomes as the spindles re-assembled over time. Un-chilled cells were also examined for the steady state microtubule length. The length of the four longest microtubule bundles emanating from the centrosome was measured to obtain the average microtubule bundle length in each cell. QLT-0267 treatment clearly slowed the rate of microtubule regrowth at the centrosome (Figures 5A and 5B). After 10 minutes of recovery at 37°C, the length of microtubule bundles in control cells reached that of steady state. By 20 minutes of recovery, overall spindle morphology resembled the steady state spindles. In contrast, QLT-0267-treated cells had very short microtubule bundles after 10 minutes of regrowth and took up to 20 minutes to reach steady state length.

Since microtubule regrowth was slower with QLT-0267 treatment, we wondered if the spindle microtubules were also less dynamic. Alpha-tubulin becomes acetylated over time [36] and this acetylated state can be used as a marker for more stable, long-lived microtubules in the mitotic spindle. We quantified the levels of α-tubulin acetylation relative to total α-tubulin in control and QLT-0267-treated HeLa cells (Figures 5C–D). Consistent with the view that tubulin acetylation occurs in the more stable kinetochore fibers [37], the level of α-tubulin acetylation in control cells was very low in prometaphase (when the spindle has not been fully organized), and became more prominent by metaphase (Figure S3). Despite the fact that the overwhelming majority of QLT-0267-treated cells were unable to form typical metaphase configuration and appeared arrested in a prometaphase-like stage (Figure 5C, bottom panel; also seen in Figure 5A, lower panel, “steady state” condition), the relative intensity of acetylated-α-tubulin in QLT-0267-treated spindles was much higher than that of prometaphase control cells (Figure 5D), and was comparable to metaphase control cells (Fig. S3). This indicates the presence of more stable kinetochore and/or non-kinetochore microtubules in the disorganized spindles of QLT-0267-treated cells. This result is consistent with our interphase microtubule dynamics data that shows inhibition of ILK by QLT-0267 leads to more stable microtubules, suggesting that ILK has similar roles on microtubule dynamics during interphase and mitosis.

Taken together, QLT-0267 treatment suppresses microtubule dynamics possibly through inhibition of ILK activity. Since ILK activity is required for formation of the Aurora-A/ch-TOG/TACC3 complex in mitosis [10], these results are consistent with previous reports that depletion of ch-TOG [12] or TACC3 [13] leads to slower microtubule regrowth. In addition, ch-TOG depletion has been shown to result in increased acetylation of α-tubulin in the mitotic spindle [38]. Interestingly, another ILK-regulated protein, β-catenin [20], was reported to localize to the centrosome, is essential for establishing a bipolar mitotic spindle [39] and also for regulating microtubule regrowth [40]. It is likely that ILK activity regulates spindle microtubule dynamics through its influence on the ch-TOG/Aurora-A/TACC3 complex and possibly other centrosomal proteins such as β-catenin. However, despite being an ILK-selective small molecule inhibitor, we cannot rule out the possibility of off-target effects of QLT-0267 on other mitotic kinases such as polo-like kinases or Aurora kinases, which have not been tested before.

### Conclusion

Our data presented here suggest that ILK overexpression, found in many types of tumors [5], destabilizes microtubules and could be one of the mechanisms behind decreased paclitaxel sensitivity of tumors. The finding that inhibition of ILK results in the opposite, i.e. microtubule stabilization, demonstrates a direct role of ILK in regulating microtubule dynamics. It will be useful to further investigate relationships between ILK overexpression and paclitaxel resistance in clinical samples, and also to explore any possible effects of ILK overexpression on the spindle assembly checkpoint.

## Materials and Methods

### Antibodies, drugs and small molecule inhibitor

The following primary antibodies were used for immunofluorescence and western blot: mouse DM1A anti-α-tubulin (Sigma-Aldrich, Oakville, Canada), rabbit anti-α-tubulin (GeneTex, Irvine, USA), mouse anti-acetylated-α-tubulin (acetylated Lys40) (Sigma-Aldrich, Oakville, Canada), human anti-centromere (Antibodies Inc., Davis, USA), rabbit anti-pericentrin (Abcam, Cambridge, USA), mouse anti-ILK (BD Biosciences, San Jose, USA), XX anti-GAPDH () and rabbit anti-flag (Cell Signaling Technology, Danvers, USA). AlexaFluor 488 anti-mouse and AlexaFluor 594 anti-rabbit (Invitrogen, Carlsbad, USA) were used as secondary antibodies for immunofluorescence staining, while IR-Dye 680-conjugated anti-rabbit and IRDye 800-conjugated anti-mouse antibodies (Rockland Immunochemicals, Inc., Gilbertsville, USA) were used for western blotting.

The small molecule inhibitor, QLT-0267, was a kind gift from Quadra Logic Technologies Inc. (Vancouver, Canada), and was dissolved in dimethyl sulfoxide (DMSO). Paclitaxel (Sigma-Aldrich, Oakville, Canada) and nocodazole (Sigma-Aldrich, Oakville, Canada) were also dissolved in DMSO.

### Cell culture, treatment, lysis and cell viability assay

HeLa cells were grown in DMEM, supplemented with 10% fetal bovine serum, at 37°C in 5% CO_2_ atmosphere. Subsequent stable cell lines derived from the parental HeLa cells were cultured in the same manner. Unless otherwise stated, drug treatment with QLT-0267 was carried out at 10 µM for 6 hours under regular cell culture conditions. Nocodazole treatment was performed at 1 µM for 16 hours. Cells were treated with varying concentrations of paclitaxel dissolved in DMSO under regular culture conditions for 48 hours. Cell viability was determined using the cell proliferation kit I (MTT) from Roche (Basel, Switzerland) that utilizes the MTT (3-(4,5-dimethythiazol-. 2-yl)-2,5-diphenyl tetrazolium bromide) assay. For microtubule regrowth experiments, cells were treated with DMSO or QLT-0267 for 6 hours, under regular culture conditions, but were placed on ice at 4°C for the last hour of treatment. The cold media was replaced with pre-warmed media containing the same drug concentration and the cells were returned to 37°C for the indicated times before methanol fixation. Cells were lysed with an NP-40 buffer containing 50 mM Tris (pH7.6), 150 mM NaCl, 1% NP-40, and 1 mM ethylenediaminetetraacetic acid (EDTA), supplemented with 1× Protease Inhibitor (Roche, basel, Switzerland), 1 mM Na_3_VO_4_, 1 mM NaF and 20 mM β-glycerophosphate. Protein concentration was determined using the bicinchoninic acid (BCA) assay.

### Generation of stable cell lines

Using a previously made pIRES-hrGFP-ILK [8] as template, Flag-ILK was amplified, by PCR with primers that added an XhoI site plus a kozak sequence at the 5′ end (ILK-XhoI-For: 5′-GGACTCTCGAGGCCATGGACGACATTTTCACTCAGT-3′) and an XbaI site at the 3′ end (ILK-XbaI-Rev: 5′-GACTTCTAGATGCAGTCGTCGAGGAATTGCTAT-3′). The resulting PCR product was cloned into XhoI- and XbaI-digested lentivirus pLVX-puro vector (Clontech, Mountain View, USA) to generate lentiviral construct pLVX-puro-ILK. This construct, together with pLVX-puro (empty vector, EV), was individually packaged into lentivirus particles in 293T cells (American Type Culture Collection) using lentiviral packaging mix (Sigma, Oakville, Canada). HeLa cells were infected with these lentivirus particles and selected in culture media containing 3 µg/mL puromycin for two weeks. Single cell-derived stable cell lines were further obtained by culturing cells in a 96-well plate using a cell sorter. The stable cell lines were designated as Vector 6, Vector 8, ILK 7 and ILK 10.

Using CAG_Tubulin5_VEN (kind gift from Dr. Peter Lansdorp) as a template, the N-terminal half and C-terminal half of Tubulin-Venus were amplified by PCR with primer pairs for the N-terminus (N-EcoRI-Fwd: GCCTGAATTCACCATGTTCATGCCTTCTTCTTTTTCCT; N-BamHI-Rev: GAGCTTGTTGGGGATCCATTCCACGAAGTAGCTGCT) and the C-terminus (C-BamHI-Fwd: GCAGCTACTTCGTGGAATGGATCCCCAACAATGTCAA; C-BamHI-Rev: GACAGGATCCTTACTTGTACAGCTCGTCCATGCCGAGA), respectively. The N-terminal half of Tubulin-Venus was cloned into EcoRI- and BamHI-digested pLVX-IRES-Neo (Clontech, Mountain View, USA), generating the construct pLVX-Neo-N-terminus. The C-terminal half of Tubulin-Venus was then cloned into BamHI-digested pLVX-Neo-N-terminus, resulting a construct (pLVX-Neo-Tubulin-Venus) that contains the full length of Tubulin-Venus fusion protein. As described above, lentivirus particles prepared with pLVX-Neo-Tubulin-Venus were used to infect Vector 8 and ILK 10. These infected cell lines were then selected in culture media containing 600 µg/mL G418 for two weeks, and populations of cells with similar levels of fluorescence at 488 nm were selected on a FACSorter (BD Biosciences, San Jose, USA).

### Immunostaining and image analysis

Cells were fixed with 100% cold methanol at −20°C for 10 minutes and stained as previously described [10]. Viewing of fixed cells and image acquisition were performed with a Zeiss Colibri fluorescence microscope, equipped with AxioCam MRm camera and AxioVision Rel. 4.8 software (Carl Zeiss AG, Oberkochen, Germany). Z-stacks at 0.25 µm intervals were acquired for analyzing centromeres and mitotic spindles. The number of slices was accommodated so that the depth of the entire mitotic cell and all centromeres were captured in the stack. In order to more clearly visualize centromeres and mitotic spindle bundles, the z-stacks were deconvolved using AutoQuant ×2 (Media Cybernetics, Bethesda, USA). For qualitative analyses, maximum intensity projection of each z-stack was used. For the measurement of mitotic spindle bundle length in the microtubule regrowth experiments, images were viewed at maximum intensity projection, and the length measurement tool in AutoQuant ×2 was used to measure the length of the four longest mitotic spindle bundles in each cell. For the measurement of inter-centromere distance, individual z-stacks of the deconvolved images were visually analyzed to pick out identifiable pairs of centromeres localized in the same focal plane. For quantification of acetylated α-tubulin signal intensity, 13 z-stacks at 1 µm spacings were obtained. The images were not deconvolved to avoid alteration to signal intensities. The stacks were viewed in ImageJ (http://rsb.info.nih.gov/ij/) at maximum intensity projection, and background was subtracted using a rolling ball radius of 50, before measuring the integrated density within a fixed round area drawn around the spindle.

For mitotic index, cells were imaged for Hoechst 33342 and anti-α-tubulin stains with 10× or 20× objective. The number of mitotic cells that contained spindles and the total number of cells based on the DNA stain were manually counted using ImageJ. The mitotic index was then presented as a percentage of mitotic cells over the total number of cells in the image.

### Live cell imaging and image analysis

Live cell imaging for measuring duration of mitosis was carried out on a heated microscope stage at 37°C in 5% CO_2_ using a Zeiss Colibri microscope (Carl Zeiss AG, Oberkochen, Germany). Phase images were acquired on an AxioCam Mrc camera every 2 minutes for 10 hours. The contrast and brightness of images were adjusted in AxioVision Rel. 4.7 to better visualize DNA within cells. For prophase and prometaphase, the combined duration was measured, since the transition from prophase to prometaphase, marked by nuclear envelope breakdown, was hard to visualize in the acquired images. Moreover, the use of a completely rounded cell as a marker for the onset of prometaphase proved difficult since a metaphase plate was observed in many cells before they were completely rounded up (Figure S4). The appearance of a relatively thin and straight metaphase plate was set as the beginning of metaphase. Anaphase was defined as the separation of aligned chromosomes. The end of mitosis was determined by the appearance of a cleavage furrow. Due to the subjective nature of measuring the time of each mitotic stage, and to remove bias, a second researcher performed blind scoring of the duration of mitosis without knowing the identities of the cell lines and confirmed the results reported here. This researcher had not seen the cell lines prior to the scoring and could not have identified them by morphology.

For live cell imaging to measure microtubule dynamics in cells expressing tubulin-venus, 1.5×10^5^ cells were seeded onto a 35 mm glass bottom dish (MatTek, Ashland, USA) one day before imaging. Immediately before imaging, cells were washed with PBS +/+ (PBS containing Ca^2+^ and Mg^2+^) twice and replaced with 3 ml of Leibovitz's L-15 media (Invitrogen, Carlsbad, USA) containing 10% FBS and oxyrase (1∶100) (Oxyrase, Mansfield, USA). The appropriate concentration of DMSO or QLT-0267 was also included in the L-15 media. Imaging was performed with a high speed spinning disc confocal microscope (Perkin-Elmer, Waltham, USA) equipped with a heated stage and objective every 3 seconds over 3–5 minutes, and acquired using the software Volocity (Improvision, Coventry, England).

A series of time-lapse images were exported from Volocity and analyzed using ImageJ. The images were adjusted for brightness and contrast in ImageJ to achieve optimum visualization of microtubule ends. The positions of the ends at every time frame were then marked with a mouse-controlled cursor using the Manual Tracking plugin. Due to the resolution limit of a confocal microscope [41], only changes in length >0.5 µm were considered growth or shortening events. Phases of undetectable changes in length (≤0.5 µm) were considered pauses. The calculations for transition frequencies and dynamicity were as described previously [19], [28]. The rescue frequency was calculated by dividing the number of transitions from shortening to pause and shortening to growing by the time spent shortening or total distance shortened. The catastrophe frequency was calculated by dividing the number of transitions from pause to shortening or growing to shortening by the sum of the time spent growing and pausing or by the total distance grown. Dynamicity was calculated as the total length grown and shortened divided by the life span of the microtubule.

## Supporting Information

Figure S1 **Generation of HeLa cell clones that stably overexpress Flag-ILK.** Flag-vector or Flag-ILK was stably introduced into parental HeLa cells by lentiviral transduction. Single cell clones were expanded from the bulk culture and 4 clones were selected for further experimentation - two vector control clones (Vector 6 and Vector 8), and two Flag-ILK-expressing clones (ILK 7 and ILK 10). (A) The clones were fixed and immunostained with anti-paxillin and anti-flag to determine the localization of Flag-ILK to the focal adhesions (arrowed). Bar = 10 µm. (B–C) Western blot analysis of mitotic spindle fractions of the HeLa clones to confirm the (B) presence of Flag-ILK at the mitotic spindle and the (C) co-purification of Flag-ILK and α-tubulin in the purified mitotic spindle fraction (labeled P). S1 and S2 are detergent-soluble fractions and S3 is the isolation buffer-soluble fraction. GAPDH is not associated with the mitotic spindle (fraction P) and acts as a purification control. (D) Representative live cell images of the HeLa cell clones Vector 6, Vector 8, ILK 7 and ILK 10. Bar = 100 µm.
(TIF)

Figure S2 **ILK-overexpressing HeLa cells assemble mitotic spindles that appear normal.** Representative immunofluorescence images of Vector 8 (control) and ILK 10 (ILK-overexpressing) HeLa cells, that were stably transfected with venus-tubulin. Cells were fixed and stained for α-tubulin (green) to show the mitotic spindle and Hoechst 33342 (blue) to show chromosomes.
(TIF)

Figure S3 **Quantification of the integrated density of acetylated α-tubulin immunofluorescence signal relative to that of total α-tubulin.** QLT-0267-treated cells typically had a prometaphase-like appearance but showed an acetylation level closer to that of control metaphase cells than control prometaphase cells. Results represent mean ± S.E.M., (N = 15 for DMSO prometaphase, 20 for DMSO metaphase, 20 for QLT-0267) and are typical of 2 independent experiments.
(TIF)

Figure S4 **Time lapse imaging of control Vector 8 and ILK-overexpressing ILK 10 cells undergoing mitosis.** Representative time lapse images of cells that reached metaphase before completely rounding up, precluding the use of a completely rounded cell as a marker for prometaphase onset. Top panel: three different Vector 8 cells. Bottom panel: three different ILK 10 cells. Bar = 10 µm.
(TIF)

Table S1 **P values of differences observed in percentage viability between control and ILK overexpressing cells after 48 hours of paclitaxel treatment at various concentrations.**
(DOCX)

Table S2 **Summary of microtubule dynamics in control and ILK overexpressing HeLa cells.**
(DOCX)

Table S3 **Summary of microtubule dynamics in HeLa cells with DMSO or QLT-0267 treatment.**
(DOCX)

Table S4 **Comparison of statistically significant percentage changes in microtubule dynamics between ILK inhibition using QLT-0267 and ILK overexpression.**
(DOCX)

Movie S1 **Time lapse imaging of a Vector 8 cell undergoing mitosis.**
(WMV)

Movie S2 **Time lapse imaging of an ILK 10 cell undergoing mitosis.**
(WMV)

Movie S3 **Fluorescent time lapse imaging of a venus-tubulin-Vector 8 cell treated with DMSO.**
(MOV)

Movie S4 **Fluorescent time lapse imaging of a venus-tubulin-Vector 8 cell treated with QLT-0267.**
(MOV)

Movie S5 **Fluorescent time lapse imaging of an untreated venus-tubulin-Vector 8 cell.**
(MOV)

Movie S6 **Fluorescent time lapse imaging of an untreated venus-tubulin-ILK 10 cell.**
(MOV)

Text S1 **Supplementary materials and methods, and supplementary references.**
(DOCX)
