# Supplementary figures and images for: Integrin-Linked Kinase Regulates Interphase and Mitotic Microtubule Dynamics
Source: PLoS One. 2013 Jan 21;8(1):e53702. doi: 10.1371/journal.pone.0053702 (PMC3549953; doi:10.1371/journal.pone.0053702)

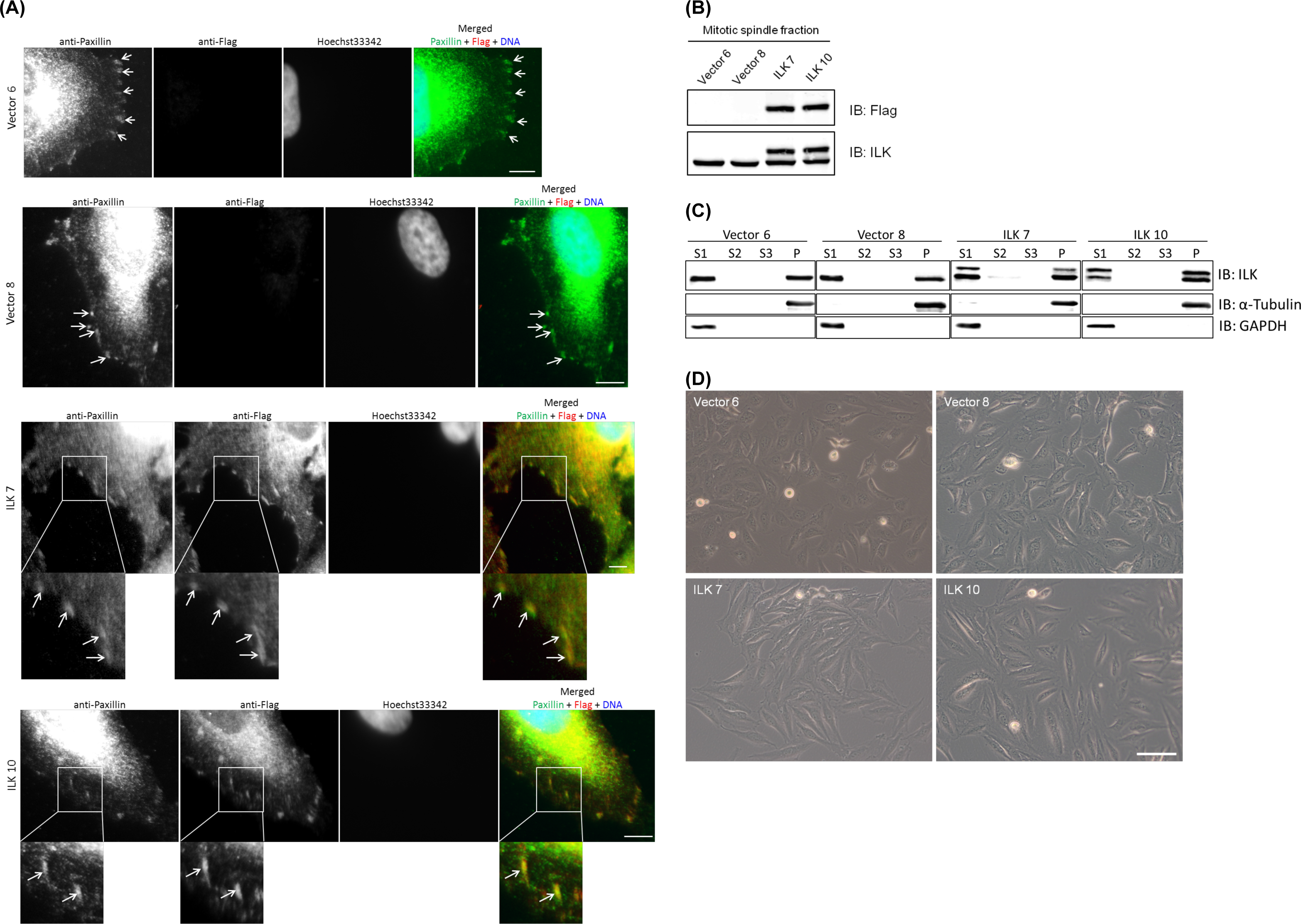

Supplement: Figure S1 — Generation of HeLa cell clones that stably overexpress Flag-ILK. Flag-vector or Flag-ILK was stably introduced into parental HeLa cells by lentiviral transduction. Single cell clones were expanded from the bulk culture and 4 clones were selected for further experimentation - two vector control clones (Vector 6 and Vector 8), and two Flag-ILK-expressing clones (ILK 7 and ILK 10). (A) The clones were fixed and immunostained with anti-paxillin and anti-flag to determine the localization of Flag-ILK to the focal adhesions (arrowed). Bar = 10 µm. (B–C) Western blot analysis of mitotic spindle fractions of the HeLa clones to confirm the (B) presence of Flag-ILK at the mitotic spindle and the (C) co-purification of Flag-ILK and α-tubulin in the purified mitotic spindle fraction (labeled P). S1 and S2 are detergent-soluble fractions and S3 is the isolation buffer-soluble fraction. GAPDH is not associated with the mitotic spindle (fraction P) and acts as a purification control. (D) Representative live cell images of the HeLa cell clones Vector 6, Vector 8, ILK 7 and ILK 10. Bar = 100 µm. (TIF) [file pone.0053702.s001.tif]

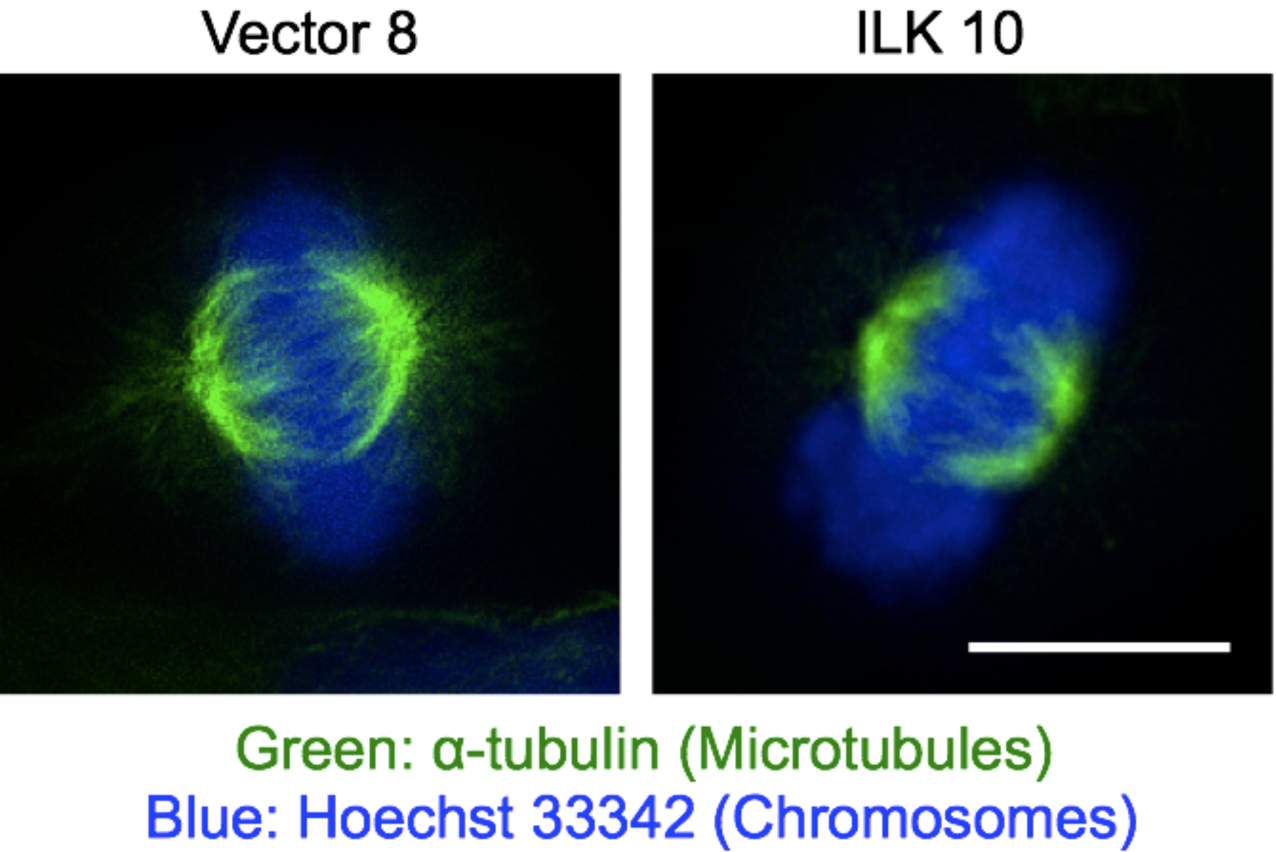

Supplement: Figure S2 — ILK-overexpressing HeLa cells assemble mitotic spindles that appear normal. Representative immunofluorescence images of Vector 8 (control) and ILK 10 (ILK-overexpressing) HeLa cells, that were stably transfected with venus-tubulin. Cells were fixed and stained for α-tubulin (green) to show the mitotic spindle and Hoechst 33342 (blue) to show chromosomes. (TIF) [file pone.0053702.s002.tif]

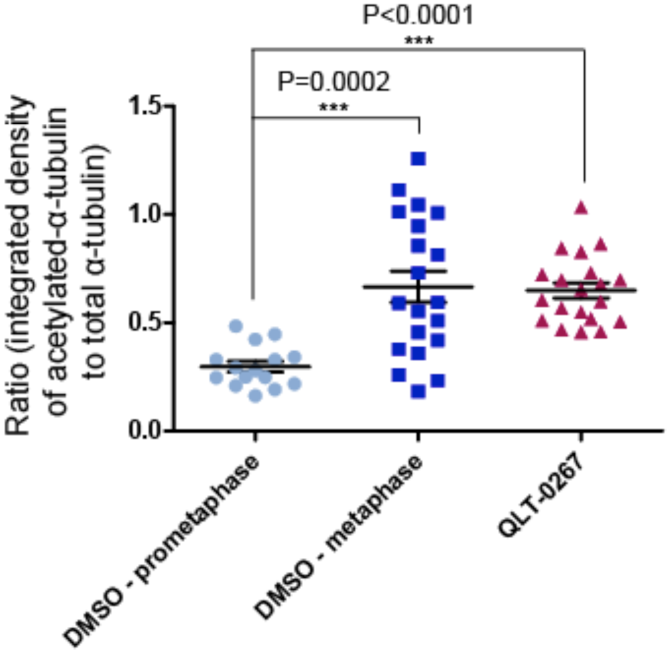

Supplement: Figure S3 — Quantification of the integrated density of acetylated α-tubulin immunofluorescence signal relative to that of total α-tubulin. QLT-0267-treated cells typically had a prometaphase-like appearance but showed an acetylation level closer to that of control metaphase cells than control prometaphase cells. Results represent mean ± S.E.M., (N = 15 for DMSO prometaphase, 20 for DMSO metaphase, 20 for QLT-0267) and are typical of 2 independent experiments. (TIF) [file pone.0053702.s003.tif]

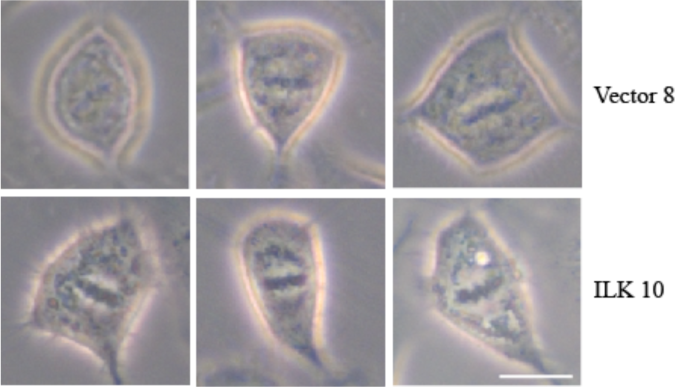

Supplement: Figure S4 — Time lapse imaging of control Vector 8 and ILK-overexpressing ILK 10 cells undergoing mitosis. Representative time lapse images of cells that reached metaphase before completely rounding up, precluding the use of a completely rounded cell as a marker for prometaphase onset. Top panel: three different Vector 8 cells. Bottom panel: three different ILK 10 cells. Bar = 10 µm. (TIF) [file pone.0053702.s004.tif]

**Suppl. Table S2: Summary of microtubule dynamics in control and ILK overexpressing HeLa cells**


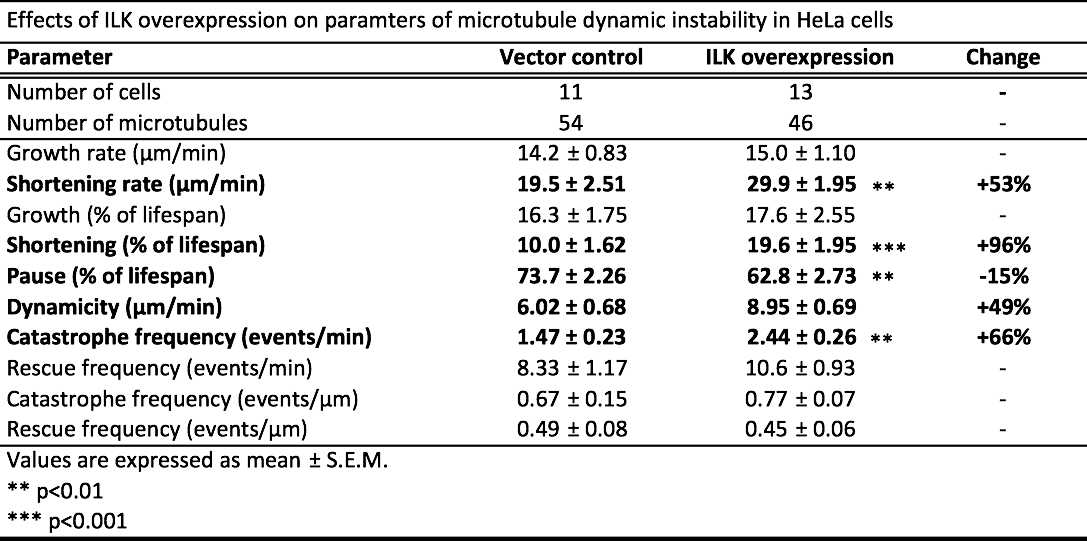

Supplement: Table S2 — Summary of microtubule dynamics in control and ILK overexpressing HeLa cells. (DOCX) [file pone.0053702.s006.docx]

**Suppl. Table S3: Summary of microtubule dynamics in HeLa cells with DMSO or QLT-0267 treatment**


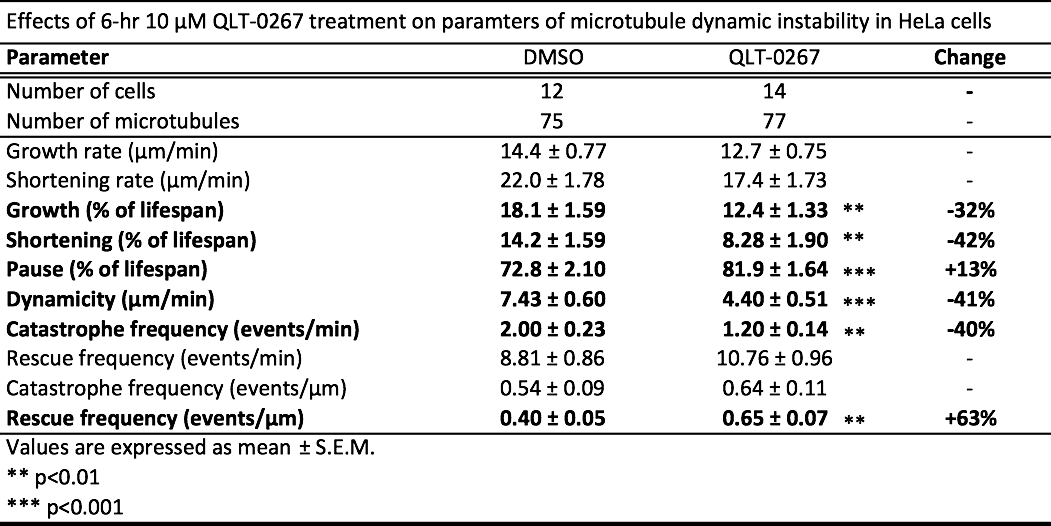

Supplement: Table S3 — Summary of microtubule dynamics in HeLa cells with DMSO or QLT-0267 treatment. (DOCX) [file pone.0053702.s007.docx]
