# Supplementary material for: Integrin-Linked Kinase Regulates Interphase and Mitotic Microtubule Dynamics
Source: PLoS One. 2013 Jan 21;8(1):e53702. doi: 10.1371/journal.pone.0053702 (PMC3549953; doi:10.1371/journal.pone.0053702)
Supplement: Table S1 — P values of differences observed in percentage viability between control and ILK overexpressing cells after 48 hours of paclitaxel treatment at various concentrations. (DOCX) [file pone.0053702.s005.docx]

**Suppl. Table S1: P values of differences observed in percentage viability between control and ILK overexpressing cells after 48 hours of paclitaxel treatment at various concentrations**

| **Cell Line** | ILK 7 | ILK 10 |
| --- | --- | --- |
| Vector 6 | [1 nM] 0.2111  [10 nM] 0.1353  [50 nM] 0.0004 ***  [100 nM] 0.0364 * | [1 nM] 0.3077  [10 nM] **<0.0001 ******  [50 nM] **<0.0001 ******  [100 nM] 0.0009 *** |
| Vector 8 | [1 nM] 0.0877  [10 nM] 0.0068 **  [50 nM] **<0.0001 ******  [100 nM] **<0.0001 ****** | [1 nM] 0.1211  [10 nM] **<0.0001 ******  [50 nM] **<0.0001 ******  [100 nM] **<0.0001 ****** |

Values presented as: [paclitaxel concentration in nM] p-value
