# Supplementary material for: Integrin-Linked Kinase Regulates Interphase and Mitotic Microtubule Dynamics
Source: PLoS One. 2013 Jan 21;8(1):e53702. doi: 10.1371/journal.pone.0053702 (PMC3549953; doi:10.1371/journal.pone.0053702)

**Suppl. Table S4: Comparison of statistically significant percentage changes in microtubule dynamics between ILK inhibition using QLT-0267 and ILK overexpression.**


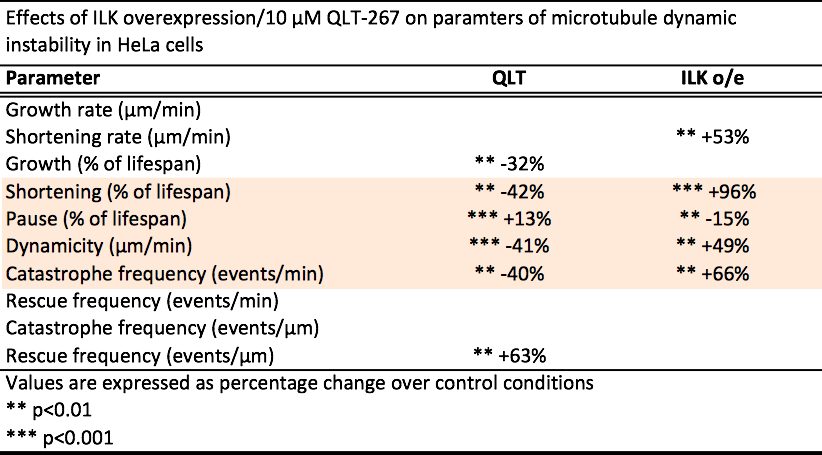

Supplement: Table S4 — Comparison of statistically significant percentage changes in microtubule dynamics between ILK inhibition using QLT-0267 and ILK overexpression. (DOCX) [file pone.0053702.s008.docx]
