# Supplementary material for: Integrin-Linked Kinase Regulates Interphase and Mitotic Microtubule Dynamics
Source: PLoS One. 2013 Jan 21;8(1):e53702. doi: 10.1371/journal.pone.0053702 (PMC3549953; doi:10.1371/journal.pone.0053702)
Supplement: Text S1 — Supplementary materials and methods, and supplementary references. (DOCX) [file pone.0053702.s015.docx]

**Supplementary Materials and Methods**

The following additional antibodies were used for immunofluorescence staining in the supplementary figures: rabbit anti-ILK (Abcam), mouse anti-paxillin (BD Biosciences). Mitotic spindles were isolated as previously described[^1^](#_ENREF_1). Images of live cells in Supplementary Figure S1D were obtained using the same microscope, camera and software as detailed in Materials and Methods. Cells were not imaged under controlled environment as the imaging took place over a very short period of time.

**Supplementary References**

1. Fielding AB, Dobreva I, McDonald PC, Foster LJ, Dedhar S. Integrin-linked kinase localizes to the centrosome and regulates mitotic spindle organization. J Cell Biol 2008; 180:681-9.
